# Structural Racism as an Ecosystem: An Exploratory Study on How Structural Racism Influences Chronic Disease and Health and Wellbeing of First Nations in Canada

**DOI:** 10.3390/ijerph20105851

**Published:** 2023-05-17

**Authors:** Krista Stelkia

**Affiliations:** Faculty of Health Sciences, Simon Fraser University, Burnaby, BC V5A 1S6, Canada; krista_stelkia@sfu.ca

**Keywords:** structural racism, First Nations, indigenous health, chronic disease, structural determinants of health, Canada

## Abstract

Indigenous peoples in Canada experience disproportionately higher rates of chronic disease than their non-Indigenous counterparts. Previous research has identified structural racism as a powerful determinant of health and wellbeing. Mounting evidence demonstrates that First Nations are disproportionately over-represented, compared to other Canadians, in several domains that have been used to measure structural racism in other countries. Despite growing concern of the impact of structural racism on health, there remains little empirical evidence on the impact structural racism has on chronic disease health outcomes of First Nations. This qualitative study examines the complex and intersecting ways in which structural racism can influence chronic disease health outcomes and the overall health and wellbeing of First Nations in Canada. In-depth semi-structured interviews were conducted with twenty-five participants, including subject matter experts in health, justice, education, child welfare, politics, and researchers in racism scholarship and First Nations who have lived experience with a chronic condition(s). Thematic analysis was used to analyze the data collected. Six themes on how structural racism influences chronic disease and the health of First Nations were identified: (1) multiple and intersecting pathways; (2) systems of failure, harm, and indifference; (3) impacts on access to healthcare; (4) colonial policies of structural deprivation; (5) increased risk factors for chronic disease and poor health; and (6) structural burden leading to individual-level outcomes. Structural racism creates an ecosystem that negatively impacts chronic diseases and the health of First Nations. The findings illuminate how structural racism can have micro-level influences at an individual level and can influence one’s chronic disease journey and progression. Recognizing how structural racism shapes our environments may help to catalyze a shift in our collective understanding of the impact of structural racism on health.

## 1. Introduction

In Canada, Indigenous peoples (First Nations, Inuit, and Métis peoples) experience disproportionately higher rates of chronic disease than their non-Indigenous counterparts, including diabetes, heart disease, cancer, tuberculosis, arthritis, and asthma [1,2,3]. A 2018 national on-reserve survey in Canada found that 59.8% of First Nations adults had at least one chronic health condition [4]. Indigenous people in Canada also have a higher overall prevalence of multimorbidity, i.e., two or more chronic conditions, compared to the non-Indigenous Caucasian population [5]. Addressing chronic disease prevalence and comorbidity is complex, as it requires the identification and examination of modifiable and non-modifiable risk factors, some of which can be influenced by social, economic, and political pathways [6,7].

There is heightened attention to the impact of structural racism on health. In 2020, the American Public Health Association declared structural racism as a public health crisis [8]. Previous research has identified structural racism as a powerful determinant of health and wellbeing and a fundamental root cause of health inequities [9,10,11,12,13]. Structural racism is defined as “the totality of ways in which societies foster racial discrimination, through mutually reinforcing inequitable systems (in housing, education, employment, earnings, benefits, credit, media, healthcare, criminal justice, and so on) that in turn reinforce discriminatory beliefs, values, and distribution of resources, which together affect the risk of adverse health outcomes.” [9]. Structural racism exists at a macro-level and is deeply imbedded and entrenched in mutually reinforcing institutional systems and societal structures [7,9]. Despite the rise in empirical studies that examine racism and health, a majority focus solely on self-reported measures of interpersonal racism, and few examine the impact of structural racism on health outcomes [7,11,14,15,16,17,18]. Studies which have examined structural racism and health outcomes have found associations in infant mortality, cardiovascular disease, and preterm births [19,20,21]. However, the impacts of structural racism on the health and wellbeing of First Nations people in Canada is underexplored.

Mounting evidence demonstrates that First Nations peoples are disproportionately over-represented, compared to other Canadians, in several domains that have been used to measure structural racism in other countries, including in health, criminal justice, child welfare, education, and the economic and labour force [22]. For example, Indigenous children account for 52% of all foster children in the child welfare system despite only representing 7.7% of children in Canada [23]. Similarly, despite representing only 4.1% of the Canadian population, Indigenous adults account for nearly one-third of admissions to Canada’s provincial and federal correction services [24]. These rates become even higher in different provinces such as Saskatchewan, where the proportion of Indigenous inmates is over seven times higher than their representation in the overall population [22,25]. There is compelling evidence of structural racism within the education system [26]. In 2016, university attainment rates for non-Indigenous Canadians were at 46% compared to 15% for First Nations peoples living on reserve and 23% for First Nations peoples living off reserve [27]. Education is a central social determinant of health and plays a critical role in shaping employment, income, and resources [28].

Furthermore, in addition to having disproportionately higher rates of chronic disease than non-Indigenous Canadians, there remain substantial gaps in life expectancy and infant mortality rates between Indigenous people in Canada and non-Indigenous Canadians [1,2,3,29]. Life expectancy at birth for First Nations peoples has been comparable to rates in developing countries and is presently 11.2 years lower than the rest of the Canadian population [2]. Across nearly all health indicators, First Nations peoples are consistently ranked as having poorer health outcomes compared to other Canadians. The over-representation of First Nations peoples in negative statistics and their under-representation in positive statistics across multiple domains are symptoms of structural racism within Canadian society [22].

Colonization has and continues to have detrimental impacts on the health and wellbeing of Indigenous peoples [30,31]. The structural inequities that continue to be witnessed are heavily shaped by the long-term historical impacts of colonialism, forced displacement and segregation, cultural genocide, and harmful colonial policies such as the pass system, the residential school system, and the Sixties Scoop [30,32,33]. This colonial environment has created an interlocking system of oppression that shapes the everyday life experiences of Indigenous peoples and has laid the foundation for structural racism to seep into every system, sector, and domain within Canadian society. Given the health inequities that continue to exist within First Nations populations, there is a critical need to understand the complex and intersecting ways in which structural racism impacts chronic disease health outcomes and the overall health and wellbeing among First Nations in Canada.

## 2. Methods

### 2.1. Study Design

The study was designed to explore multiple viewpoints and perspectives of subject matter experts (SMEs) in various domains and those with lived experience of chronic disease to uncover the complex and intersecting ways in which structural racism influences the health and wellbeing of First Nations in Canada. Data for this study were gathered from in-depth, semi-structured interviews with 25 participants, including SMEs in health, justice, education, child welfare, politics, and researchers in racism scholarship and First Nations who have lived experience with a chronic condition(s) (FNLECC). The interviews focused on participants’ perceptions, experiences, and beliefs of how structural racism influences the health of First Nations.

### 2.2. Research Setting

The research setting was based within the geographical boundaries of Canada. While the primary location was in the province of British Columbia, participants who were interviewed worked and lived in various geographical locations across Canada, spanning multiple provinces and territories. As data collection was predominantly conducted during the COVID-19 pandemic, interviews were conducted remotely by telephone and video conference.

### 2.3. Recruitment

Nonprobability purposive and convenience sampling were used to recruit participants who met the inclusion criteria. SMEs needed to have knowledge, expertise, or experience working in one or more specific target domain(s). FNLECCs needed to self-identify as First Nations and be currently living with a chronic condition or multiple chronic conditions.

#### 2.3.1. SMEs

SMEs in the field of health, justice, education, child welfare, and politics were identified by reviewing literature on the research topic to identify potential participants with knowledge, expertise, and experience in a specific domain relevant to structural racism in Canada. Participants were identified based on their expert skills, knowledge, and experience within their respective fields. Furthermore, during the recruitment phase of this study, some participants were unable to participate in the study and made recommendations for individuals they believed would hold pertinent expertise in the specific domain being requested. Contact information for participants was obtained from publicly available sources, primarily through university, government, or non-profit websites. Once contact information was obtained, an introductory email was sent to prospective participants inviting them to voluntarily partake in the study. The email included details on the purpose of the research study, estimated time to participate, number of questions to be asked, and contact information of the principal investigator for any questions regarding the study.

#### 2.3.2. FNLECCs

FNLECCs were recruited with the assistance of a First Nations community organization (FNCP) located on Vancouver Island in British Columbia, Canada. The FNCP is an organization that provides a variety of health, education, child and family, and economic programs and services to First Nations communities. The lead researcher worked in collaboration with the FNCP to identify participants for this study. As the recruitment for this study was conducted during the COVID-19 pandemic, in-person community engagement and recruitment were not advised. Therefore, the FNCP identified and referred participants who were interested in participating in this study to the principal investigator. Once identified, an email invitation was sent to each participant as an introduction to the study and to request their participation in the study given their experience, knowledge, and lived experience.

### 2.4. Participants

Overall, 25 participants were interviewed. The primary set of data was derived from interviews with 22 SMEs in seven main areas of expertise, including healthcare (*n* = 6), justice/legal (*n* = 3), politics/government (*n* = 3), education (*n* = 3), child welfare (*n* = 2), media (*n* = 1), and racism scholarship (*n* = 4). Several participants were SMEs in two domains, for example, being an active physician in the healthcare system while also being an academic scholar in the area of racism scholarship. Therefore, several participants brought an intersectoral and multi-domain perspective to this study. To complement the perspectives of the primary subject matter expert participants, three in-depth interviews were conducted with FNLECCs.

Participants were located in various geographical regions across Canada spanning the Pacific Region (British Columbia), Prairie Region (Alberta, Saskatchewan, Manitoba), Central Region (Ontario, Quebec), Atlantic Region (New Brunswick, Nova Scotia, Prince Edward Island, Newfoundland), and Northern Region (Yukon, Northwest Territories, Nunavut). While the study included participants from each region in Canada, a majority of participants were located in the Pacific region (*n* = 9) and Prairie region (*n* = 9), compared to the Central region (*n* = 5), Northern region (*n* = 1), and Atlantic region (*n* = 1). Furthermore, 80% (*n* = 20) of participants in this study self-identified as Indigenous (19 First Nations participants and 1 Métis participant) and 20% (*n* = 5) identified as being of non-Indigenous/settler ancestry. Many of the SMEs who participated in the study provided perspectives and knowledge of being a First Nations SME, along with their own lived experience.

### 2.5. Data Collection

Data were collected through in-depth, semi-structured interviews with 25 participants conducted between November 2019 and August 2020. Given the geographical location of many of the participants, along with social distancing measures related to the COVID-19 pandemic, all interviews were conducted via telephone or video conference. Interviews were conducted at a date and time that was most preferred by each of the participants. Separate interview guides were developed for SMEs and FNLECCs. Interview questions were developed by the principal investigator, a First Nations researcher, and reviewed by a supervisory committee which is composed of both Indigenous and non-Indigenous settler scholars in the area of Indigenous health to ensure cultural safety in the framing of the questions. While each had similar questions, they were adapted to be more specific to the context of each participant’s population group. Each included a series of twelve open-ended questions that began with introductory questions and moved into more detailed questions designed to elicit responses related to the overarching purpose of the study and aligned with the research questions.

For SMEs, the interviews focused on exploring participants’ views, perspectives, and opinions on how they perceived structural racism as impacting chronic disease and the overall health and wellbeing of First Nations peoples. Furthermore, questions around what they perceived as being needed to address structural racism, along with what potential domains and indicators of structural racism may be, were asked. Interviews with FNLECCs focused on exploring participants’ views about their chronic disease journey, including diagnosis, treatment, and quality of care. Furthermore, interviews focused on creating space to understand how participants viewed and understood the causes of and risk factors for their chronic disease and how their chronic condition has impacted their overall quality of life. Interviews were audio-recorded and ranged from forty-five minutes to two and a half hours in length.

### 2.6. Data Analysis

The audio-recorded interviews were transcribed verbatim and then anonymized by removing any personally identifiable information. Data were imported into NVivo12, a qualitative data analysis software program, for thematic data analysis. This study employed a seven-stage process for conducting a thematic analysis of the data that was informed by Kuckartz’s [34] framework for thematic qualitative text analysis and Braun’s and Clarke’s [35] phases of thematic analysis. A concept-driven (deductive) and data-driven (inductive) approach was utilized to develop thematic categories [36]. Main thematic categories were identified using a concept-driven approach derived from the research questions. Once the main thematic categories were developed, the first round of coding was conducted on the entire data set. The next stage involved reviewing each of the text passages that were coded to the main thematic categories to identify and generate emerging data-driven (inductive) codes. During this process, all of the text passages coded into the main thematic categories were compiled and reviewed multiple times. In reviewing the text passages, open coding was conducted, and more descriptive secondary codes were derived from the data. Once the final list of secondary codes was created, all transcripts were reviewed again and coded based on the new coding structure. A preliminary synthesis of findings was created which showcased the thematic findings in context of the research questions. The preliminary findings were shared with six participants, four SMEs and two FNLECC, as part of a member check. Sharing preliminary findings with participants created an opportunity to gather feedback and input on the preliminary findings and help to validate the themes that emerged from the analysis. Feedback from the member check interviews was incorporated into shaping the final framing and representation of the findings.

## 3. Results

Six themes emerged on how structural racism influences chronic disease and the health of First Nations in Canada: (1) multiple and intersecting pathways; (2) systems of failure, harm, and indifference; (3) impacts on access to healthcare; (4) colonial legislation and policies of structural deprivation; (5) increased risk factors for chronic disease and poor health; and (6) structural burden leading to individual level outcomes. Table 1 below provides a breakdown of the themes and subthemes.

### 3.1. Theme 1: Multiple and Intersecting Pathways

For a majority of the participants, structural racism was viewed as having a substantial impact on the health and wellbeing of First Nations peoples. Structural racism was described as influencing chronic disease and health through multiple and intersecting pathways that are embedded within interlocking systems and domains. Participants expressed how it is difficult to identify a direct causal link between structural racism and chronic disease:

*“Structural racism influences chronic disease in really complicated and convoluted ways. I think it’s hard to draw a direct link … But there’s an overwhelming number of links between why people are living with their chronic diseases and all of the pieces of structural racism”*

(SME 13)

*“It is not a simple linear relationship … It’s a complex relationship in that your health and wellbeing is undermined by multiple intersecting forms … In my mind, there’s no question the intersection between, or the causal links between, violence and trauma that Indigenous people have experienced and continue to experience and the high rates of diabetes, the high rates of hypertension, the higher rates of arthritis and the chronic pain”*

(SME 8)

The mechanisms by which structural racism negatively influences chronic disease and health was viewed to be complex, and a simplistic approach to describing its impacts would not suffice. This complexity was discussed as being one reason why structural racism is so challenging to identify and mitigate, as its roots have been firmly established across multiple domains and sectors.

#### 3.1.1. Web of Inequities and Interacting Systems of Burden

Central to the discussion on how structural racism influences chronic disease was how the web of inequities and burden often faced by First Nations peoples in their everyday lives results in their health not being prioritized over other critical issues in their life. Many participants discussed the layers of inequities that plagued many Indigenous communities, resulting in there being multiple challenges to face. As further described by one participant:

*“People are stressed because it’s coming from all different directions. So you have people who could be dealing with diabetes, could be dealing with some chronic disease, but are also dealing with the stress of two or three or four different systems that are impacting their lives or their loved one’s lives all at once, all the time.”*

(SME 16)

The example illustrates the compounding effects of structural burden on an individual’s life. It the intersection of a complex weight of inequities and being faced with multiple systems of burden that makes it challenging and nearly impossible to live a healthy life.

#### 3.1.2. Compounding Weight

Participants discussed how the continued exposure and experience of structural racism across multiple domains can result in the experience of compounding weight, which eventually begins to degrade one’s health. Several participants described the “crushing weight” of these inequities on an individual’s life as being compounding and, eventually, among other inequities, resulting in both short- and long-term poorer health outcomes:

*“this invisible heavy weight of racism that just sits on our shoulders in Canada that is so heavy, but we don’t even realize it’s there until we get out of this environment, this environment of structural racism in all areas of life. It just sits on your shoulders and weighs you down.”*

(SME 10)

The concept of weight was discussed by many participants as a way to articulate the impact of structural racism on the lives of individual people. It was viewed as not only impacting access to opportunities, including employment and education, but also the daily experience of going shopping and walking down the street. The totality of the burden that is created by an environment infused with structural racism was strongly viewed by participants to influence the health of First Nations peoples. The ‘compounding weight’ as a result of a multitude of intersecting burdens was thought to play a substantial role in the degradation of health and wellbeing and eventually result in poor health and increased risk for chronic disease. These intersecting forms of burden were also viewed as one reason why prevention efforts can often be ineffective when dealing with individuals who are facing the greatest levels of inequities in society, as illuminated by one participant:

*“How can you bring prevention when your life is in chaos, whether it’s domestic violence, overcrowding in the home, food insecurity or there’s sexual abuse? … how can you do prevention in that chaos? And then patients are chastised … instead of looking at it from the patient’s perspective who’s already overwhelmed. So, they really need to look at what prevention means and if it’s possible. Otherwise, you’re victim blaming, you’re blaming a lifestyle that the patient has.”*

(SME 7)

### 3.2. Theme 2: Systems of Failure, Harm, and Indifference

#### 3.2.1. Failure by the System to Help

Structural racism was viewed to impact health through multiple systems of failure, harm, and indifference often experienced by First Nations when engaging with systems for help. Participants expressed how many essential service institutions, which are legally mandated to provide care, treatment, or service, are often places where First Nations peoples experience harm, indifference, or denial of service. The systemic failure by multiple systems to provide appropriate care in times of need was viewed to have a negative impact on First Nations’ health and chronic disease. The case of Tina Fontaine, a First Nations teen whose death and murder in Winnipeg, Canada, remains unsolved, was referenced by participants as an example to highlight how systems which are legally mandated to provide care often fail First Nations and can often end up being detrimental to their health and lives:

*“A case like Tina Fontaine, who was a child in need of protection explicitly, understanding the number of systems she interacted with that completely failed her and were also part of why she needed multiple systems. So her involvement with CFS [Child and Family Services] and justice is what led to her need for interaction with the healthcare system. But she, as like many Indigenous peoples, can’t count on the healthcare system for help even when that is their last resort, right? … I think that can be hard for actors within the healthcare system to come to grips with … understanding or seeing that for Indigenous people, there often just another system where danger can be encountered or harm can be encountered.”*

(SME 1)

*“I think that’s a perfect example of the structural negligence that exists, where you have multiple systems which are set to out to support such individuals, but somehow Indigenous peoples still fall through the cracks.”*

(SME 21)

Participants discussed how if help and care were received when accessing these systems, then it may result in life-sustaining and health-promoting access to preventive treatment and care. Instead, this was viewed to negatively influence chronic disease outcomes and overall health and wellbeing.

#### 3.2.2. Lack of Accountability for Death and Harm

The lack of accountability in systems to hold individual actors accountable for the maltreatment or death of First Nations peoples in their care was viewed as a way structural racism influences the health of First Nations. Within the healthcare system, participants expressed how it is rare to see health professionals held accountable for harm, substandard care, misdiagnosis, or inhumane treatment of Indigenous patients. Lack of accountability was interpreted as a lack of disciplinary action, consequences, or deterrence for engaging in these types of harmful practices or actions. In particular, the lack of oversight and accountability mechanisms for regulatory bodies in investigating complaints of substandard care or mistreatment was one way in which a lack of accountability in systems to adequately address harm perpetrated against First Nations exists. For example, one participant highlighted the self-regulation of health professions as part of the reason why patient complaints of substandard care rarely result in any meaningful accountability or outcome for the patient:

*“There is a lack of oversight bodies in the system and it includes the self-regulation of health professionals. We’re self-regulated and it really does not work as the system takes care of its own”*

(SME 7)

Lack of accountability to address substandard care, harm, mistreatment, and being denied treatment was one pathway by which structural racism influences health, as it fosters an environment in which harm to First Nations goes unpunished.

Participants discussed a general unwillingness of First Nations patients to come forward to report substandard care or file a complaint against a health professional for fear of losing access to essential services. For example, due to the limited number of service providers in rural and remote areas, participants shared how many First Nations peoples were unwilling to report negative experiences or substandard care when accessing healthcare services for fear of losing complete access to essential services. Even when a complaint is filed, an FNLECC shares how little the treatment she received changed after filing a formal complaint with the health professional’s regulatory body:

*“I did get an apology from the province, however, on the ground it does not change anything and it doesn’t work for where the patient is. Everybody still gets caught up in thinking I’m blowing the whistle, I’m angry. You know? And it shouldn’t be that way.”*

(FNLECC 2)

The repercussions faced by patients who report misconduct are sometimes viewed as far greater than the actual outcome of the complaint.

### 3.3. Theme 3: Impacts on Access to Healthcare

Structural racism was viewed to impact access to healthcare through three pathways, including being denied treatment or dismissed, an unwillingness to access care due to fear and distrust in the healthcare system, and the lack or limited access to healthcare and preventative treatment.

#### 3.3.1. Denied Treatment or Dismissed

Participants frequently discussed how it was common to see First Nations denied treatment, receive lower quality of care, or have their concerns dismissed when accessing healthcare. For example, being dismissed or not having their pain recognized as valid was a shared experience among the participants living with chronic conditions. This experience was also identified by many of the SMEs in the study who either witnessed or were aware of these frequent experiences:

*“The number of people whose physical health is not taken seriously because they’re presumed to be attention seeking … is also an issue. One of the inquests that’s about to be launched into a woman’s death who was deemed that her attempts to get attention and to try and get support in the systems were all about getting attention. And then when she died, it was deemed that she had a heart attack and in fact, had there been an intervention when she first started asking for help, she likely would not have died, it could have been caught before it was a full blown heart attack. Instead, … the presumption was that she was trying to get attention or she was drug seeking instead of recognizing those symptoms as symptoms that should be taken seriously”*

(SME 11)

While being dismissed or denied treatment was discussed as being commonly viewed as a result of interpersonal racism or a few ‘bad apples’ in the healthcare system, participants highlight how the systematic exclusion of First Nations peoples from the healthcare system is one of the ways in which structural racism maintains and reinforces health inequities and impacts chronic disease.

While there was recognition by many participants that structural racism existed in the healthcare system and played an influential role in impacting the health of First Nations by limiting access to quality care, prevention, diagnosis, or treatment, it was viewed as difficult to publicly challenge due to its pervasive and also illusive nature in which it manifests, as shared by one participant:

*“It’s difficult to prove because attempts to be seen in more of the omission, the less quality for First Nations people in a healthcare setting. For example, First Nations in the Northwest Territories refer to it as the ‘invisibility complex’. When they walk into an emergency room, people don’t see them. It’s really difficult to measure and then address because it tends to be, structural racism in healthcare tends to be [identified as] less quality”*

(SME 14)

Participants described how being denied treatment or dismissed was so commonly experienced by First Nations and their families that it eventually results in fear and distrust in the healthcare system, leading to an unwillingness to access such unsafe environments.

#### 3.3.2. Fosters Fear and Distrust in the Healthcare System: Will Not Access

Structural racism was viewed to impact healthcare access by fostering fear and distrust in the healthcare system, thus leading to First Nations not feeling safe enough to engage with the system. For example, participants expressed how the widespread and systemic experience of First Nations being denied treatment or dismissed when accessing healthcare can result in fear and distrust to seek medical treatment and care. The historical mistreatment and exclusion of First Nations peoples from the healthcare system was also viewed as having direct linkages to present-day fear and distrust in accessing healthcare. Participants expressed how the long-term impacts and intergenerational trauma experienced by First Nations in Indian hospitals, tuberculosis sanitoriums, medical experiments conducted in residential schools, and the segregated healthcare system that existed in Canada have unequivocally destroyed First Nations peoples’ trust in the Canadian healthcare system. One participant contextualizes how fear and distrust in the healthcare system from historical harms can have adverse effects on present-day health outcomes:

*“We see a distinct lack of trust in a lot of Indigenous populations to access healthcare and how scary health institutions are to them. That fractured relationship is longstanding, in terms of that distrust … So when you’re talking about structural racism, it can’t but impact overall health and wellness because you need to engage in a system in order to receive benefit from it. And if you’re in a place where there’s such distinct mistrust, because you’ve been abused by that system for so long, then there’s no benefit that can be obtained.”*

(SME 20)

Experiences of racial discrimination and stereotyping when accessing care were also raised as a reason why First Nations may choose to disengage from the healthcare system despite requiring lifesaving, medically necessary care. For example, one participant shared her experience on a study which examined HIV rates in a Canadian province:

*“We looked at why First Nations aren’t accessing services and racism was one of them. How they’re treated at places when they walked through the doors of different agencies or places where they should be provided with care, treatment and support, however, because of how they have been treated, they won’t walk through there. So we have people walking around that have never been tested because they won’t walk into a testing place. They’ve never been treated, they don’t take HIV meds because of how they’ve been treated”*

(SME 6)

Previous experiences of racial discrimination when accessing care or feeling unwelcome, excluded, or judged were viewed to be one way in which structural racism, through its manifestation in interpersonal racism, impacts chronic disease and overall health and wellbeing.

The unwillingness to seek medical treatment due to the risk it may pose to their family and the potential risk of exposure to other harmful systems was also identified as a source of fear and distrust in the healthcare system. For example, one participant describes how she views structural racism impacting healthcare access through the pathway of the child welfare system:

*“child welfare being a big determinant for a lot of Indigenous women in terms of fear of accessing healthcare for themselves in case there are medical conditions, particularly if there is any fear related to mental health will be used to remove the children from their care.”*

(SME 18)

The dilemma to access emergency care or risk having their children taken away by child and family services was a lived reality that was shared by participants living with a chronic condition. Fear that their health may be perceived by healthcare staff to impact their ability to take care of children and the potential risk of child welfare taking their children away was a direct deterrent for accessing healthcare.

#### 3.3.3. Lack of or Limited Access to Healthcare and Preventative Treatment

Structural racism was also viewed to influence chronic disease through the lack of or limited access to healthcare and preventative treatment. The limited healthcare capacity available within many First Nations communities and the geographical distance to urban centres where specialized services could be accessed was viewed as a major barrier to accessing the preventative and emergency care required for maintaining optimal health and wellbeing:

*“the lack of resources available to First Nations especially when they’re in isolated areas. You look at access to a physician or a dentist, to the lab, to breast screening, to a lot of the pre-requirements for health and they don’t have access to them … they need to go to a specialist, they need to go to surgery, they need to go to healthcare they require that’s not available at the community”*

(SME 7)

The limited access to preventative healthcare was seen as detrimental to the long-term health of First Nations and contributed to the progression of diseases that could have been treated early on before progressing into more serious health issues:

*“there’s everything from diabetes, to heart disease, to cancer to other illnesses … there are countless examples where if those had been dealt with in a more preventative way, people would not have progressed to the state that they did”*

(SME 11)

The limited availability of healthcare which incorporates Indigenous knowledge and worldviews was also identified by participants as another way structural racism influences healthcare access. Participants shared how mainstream systems continue to privilege Western biomedical paradigms and knowledges while dismissing Indigenous ways of knowing and being, which has implications for First Nations living with chronic disease:

*“Western knowledges are so privileged in the health system … Indigenous approaches to health and wellness are often erased, not present at all, or disregarded or assumed to be mythical, mystical or not feasible. That impacts Indigenous folks primarily through every interface of the system as it relates to both acute and chronic disease, treatment, diagnosis, and ongoing wellness strategies … In the Western systems, we break things down in terms of disease states, you’ll see, chronic disease management programs for diabetes, one for depression, one for anxiety, and one for substance use. However, more often in our Indigenous communities, it’s far more of a holistic approach to health and wellness in ways that consider a whole person and not just one aspect of them.”*

(SME 13)

The privileging of Western knowledge in healthcare and medicine was perceived to be another barrier which impacts access to quality healthcare for First Nations. In the context of chronic disease treatment and care, this was discussed as being a source of how the system does not meet the needs or worldview of Indigenous patients. Several participants expressed how by seeing how their values, teachings, and knowledge were absent from standard clinical treatment and care, it fostered an unwillingness to engage with particular treatment programs.

### 3.4. Theme 4: Colonial Legislation and Policies of Structural Deprivation

Participants discussed the harmful impacts of colonial legislation and policies administered by the federal government of Canada as shaping the everyday lives of First Nations peoples and ultimately impacting chronic disease and their overall health and wellbeing. The role of colonial policies was viewed as a critical mechanism by which structural racism continues to be reinforced in Canada and shapes the social determinants of health and wellbeing of First Nations, as further described by one participant:

*“I think about all the ways that policies of exploitation and deprivation, intentional policies that have impacted and determined the broader circle of access to clean drinking water, to safe housing, educational resources, adequate food, and resources to support the wellbeing of our own family.”*

(SME 18)

Within the health domain, SME 3 shares how there is a critical need to “*address the legal structure that’s underpinning these horrific laws that keeps implementing colonialism and racism within our healthcare field*”. The structural impacts of historical and present colonial policies which are rooted in inherent racism were viewed by participants as being integral to understanding the high rates of chronic disease that exist within First Nations communities. While racism in law and policies are traditionally classified by scholars as systemic racism, however, the degree and magnitude to which it is perpetuated across domains and systems was viewed to be a manifestation of structural racism.

#### 3.4.1. Colonial Paternalism via the Indian Act: Root Causes of Chronic Disease and Poor Health

Participants frequently referenced the Indian Act as an example of colonial legislation that has played a powerful role in sustaining structural racism and oppression within Canada, primarily as it was viewed to be fundamentally rooted in the ideology that First Nations peoples are inferior. One participant shares how the Canadian government’s policies and legislation are intrinsically linked to poor health outcomes in First Nations communities:

*“The TRC’s [Truth and Reconciliation Commission of Canada] call to action 18 says that we should understand the gap in Indigenous health as a result of previous government policy … That type of population level health gap that we see most of it has to do with longstanding structures, policies, legislation, discourses that shaped the conditions we are living in … The number one determinant of which is low income and living in poverty and the entrenchment of poverty in Indigenous communities because of the impact of the Indian Act and, you know, keeping in mind that all of these policies are founded in the same underlying racist ideology that Indigenous people are less than human”*

(SME 1)

Participants described how the racist ideologies which underpin much of the current and historical laws, legislation, and policies indirectly impact present day chronic disease inequities within First Nations peoples. The degree to which structural racism is embedded and infused into all systems and sectors to be used as a colonial tool to maintain power and privilege of settlers was referenced by participants. SME 14 states, “*the roots of racism in the country are in the legislative racism that exists in the Indian Act*”. The Indian Act was raised by many participants as being central to the colonial paternalism that exists in the historical and, at times, present relationship between First Nations and the government, as expressed by one participant:

*“And that goes back to that Canadian government, that Canadian system that made sure that those provisions of the Indian Act determined every aspect of what it meant to be Indigenous and coming out of that has meant we are fighting on every front because every part of that piece of legislation controlled and destroyed our sense of self for so long.”*

(SME 20)

#### 3.4.2. Resource Allocation: Chronic Underfunding of Essential Services

Colonial policies were viewed as influencing chronic disease and health through the chronic underfunding of essential services, including the child welfare, health, justice, and education systems. Participants discussed how despite the misconception held by a segment of the Canadian population that First Nations peoples ‘get everything for free’ or are given ample amounts of resources to run on-reserve programming, the chronic underfunding of essential services by the federal government is an example of how structural racism continues to have long-term impacts on poor health outcomes and chronic disease within Indigenous communities. The essential requirements to live a healthy life, including access to clean water, healthcare, and housing infrastructure, were discussed as being so heavily underfunded that it created an environment in which First Nations could not be well. For example, one participant shared:

*“There is a structural inequity around resource allocation for most if not all of the social determinants of wellbeing for First Nations. There’s less money on the table for such things as housing for First Nations, so then that risk factor is increased. There’s less money on the table for clean water, there’s less money on the table for frontline healthcare to serve in First Nations communities. With all those combined, it just increased the risk factors especially for all sorts of wellbeing issues including chronic disease.”*

(SME 14)

The chronic underfunding of essential services by the federal government for on-reserve First Nations communities was viewed as one pathway through which structural racism manifests and has an impact on health, wherein underfunding directly impacts access to the fundamental basics required to live healthy lives.

### 3.5. Theme 5: Increased Risk Factors for Chronic Disease and Poor Health

Structural racism was viewed as influencing health by increasing risk factors for chronic disease and poor health outcomes. Participants expressed how structural racism increases one’s risk of experiencing events, lifestyles, or behaviors which are known to be associated with increased risk for chronic disease.

#### 3.5.1. Shaping Critical Social Determinants of Health

A common pathway discussed by participants on how structural racism increases risk factors for chronic disease and poor health was by shaping key social determinants of health. Instead of being viewed as a social determinant of health, structural racism was viewed by participants as influencing all social determinants of health. For example, critical social determinants required for good health, including access to quality education, employment, housing, and preventative health treatment and care were frequently discussed in the context of those living in poverty. Structural racism was seen to increase one’s risk of living in poverty, which then can have detrimental impacts on chronic disease progression and risk of poor health, as further discussed by one participant:

*“Whether it be the pre-onset of diabetes or heart conditions, if they had supports and resources earlier on, they would have had a preventable crisis instead of ending up in emergency rooms because they couldn’t afford the food, they couldn’t afford the medication, they couldn’t afford the lifestyle that would be required for them to be able to manage their diseases, heart diseases … That, to me, is one of the most clear examples of structural racism that contributes to chronic disease and overall health and wellness. The inequality because of poverty.”*

(SME 11)

Lack of access to critical social determinants of health was viewed as increasing one’s risk of being in an environment which results in poor health outcomes.

#### 3.5.2. Hyper-Surveillance: Increased Risk for Interaction with the System

Structural racism was discussed as increasing the risk for engagement with multiple systems, including the criminal justice system. For example, participants frequently perceived structural racism as influencing risk factors for chronic disease and poor health through the increased risk of incarceration experienced by First Nations. The hyper-surveillance of First Nations by police, both historically and presently, along with the over-representation of Indigenous peoples in the justice system, were viewed as critical risk factors that have long-reaching impacts on one’s health and the health of their families. A SME in the area of health shared:

*The fact that Indigenous people are more likely to be stopped, to be arrested, more likely to be charged, more likely to be convicted and more likely to receive longer sentences. Time and again, every kind of phase of the justice system. And then being incarcerated is bad for your health, including infectious diseases, including HIV … Also higher rates of mental health conditions.*

(SME 1)

Participants expressed how being at increased risk for racial profiling, engagement with systems, and incarceration has long-term connections to health. Therefore, rather than being a simple linear relationship between incarceration and chronic disease and health outcomes, structural racism, through its multiple and interfering pathways into one’s life, can influence the environment in which one lives to then increase the risk factors for First Nations to experience poor health outcomes. Therefore, rather than viewing incarceration as a discrete cause of poor health, participants spoke of the way structural racism functions as a connector of myriad influences over one’s life experiences, thus placing them in an environment that ultimately predisposes them to risk and which, in turn, may lead to health challenges.

#### 3.5.3. Colonial Burden and Trauma: Unhealthy Coping Mechanisms

Participants shared how trauma experienced by First Nations peoples, both individual trauma and collective trauma, can negatively influence lifestyle factors, unhealthy coping mechanisms, and overall risk factors for chronic disease and poor health. Participants shared how the trauma inflicted from residential school has impacted generations of First Nations families and their willingness or not to seek healthcare for their chronic conditions. The lack of trust in the healthcare system has resulted in some First Nations engaging in unhealthy self-medicating practices to avoid seeking healthcare treatment for their chronic condition. The weight of structural racism and the experiences of everyday racism was also discussed as taking a significant toll on one’s health and wellbeing, as further detailed by one SME:

*“So over time, it takes a toll mentally, psychologically, emotionally and physically. And for some people, it’s fatal … In terms of comorbidities, it’s why we deal with alcoholism, you know, lateral violence, all of those coping mechanism, those dysfunctional coping mechanisms by which the stress is internalized … So over time, I think that it has a catastrophic effect on health because racism isn’t something that you can get over. The only thing that we can do as people who experience racism is learn effective coping mechanisms. And I think that many, many people don’t cope with it effectively.”*

(SME 15)

### 3.6. Theme 6: Cumulative Structural Burden Leading to Individual Level Health Outcomes

Structural racism was discussed as negatively impacting chronic disease through the physical embodiment of the cumulative structural burden, resulting in direct physiological outcomes. Viewed by participants as one of the most direct ways in which structural racism influences chronic disease health outcomes, the embodiment of one’s experiences and circumstances manifests into physical and biological outcomes.

#### 3.6.1. Chronic Stress and Elevated Allostatic Load

Structural racism was viewed as impacting chronic disease by increasing the level of chronic stress and elevated allostatic load experienced over one’s life course, resulting in a negative biological toll on one’s body. The chronic stress triggered by multiple and intersecting burdens brought on by structural racism was viewed by many participants to eventually wear people down and result in a high allostatic load. An SME describes how the compounding weight of structural racism on people can eventually impact their biological aging and allostatic load:

*“high blood pressure creeping up, high cortisol, people are in fight or flight all the time. We’re starting to see problems with metabolism, the waist circumference is growing, we’re seeing problems with inflammation … The greater the number of situations they’re experiencing in their life from a structural perspective. It’s not just one aspect, but if it’s two aspects, three aspects, or four aspects at work. It’s really impacting the aging of their body”*

(SME 10)

#### 3.6.2. Burnout and Poor Mental Health

The impact of cumulative structural burden was often discussed by participants in reference to burnout and poor mental health implicants for First Nations peoples working in highly colonial systems. Participants shared their challenges of working in highly colonial structures, such as a university or medical settings, where they have experienced double expectations, lateral violence, and racism from colleagues. This was described as taking a heavy toll on individuals and leading to burnout and poor mental health. Within academia, one participant shared:

*“I think about the number of Indigenous women or Indigenous men, the few that we do have at the university level, and I see more folks going on leave and having mental health issues because of the stress of being in the space constantly having to be defending and advocating. And that’s the responsibility we all have right?”*

(SME 17)

Chronic stress, burnout, and poor mental health was viewed as the direct physical embodiment of experiencing the cumulative structural racism that exists across multiple systems and sectors within Canadian society.

## 4. Discussion

The findings suggest that the way in which structural racism influences chronic disease and overall health and wellbeing of First Nations in Canada is complex, multifaceted, and expressed through multiple and intersecting pathways. Rather than being a direct causal relationship, the findings suggest that structural racism operates across multiple systems and pathways to result in a compounding or crushing weight that eventually begins to degrade one’s health and facilitates an ecosystem in which poor health and chronic disease can prevail. The burden, weight, and totality of these oppressive experiences were described as having a compounding effect on one’s health across the life course and can limit or hinder access to critical social determinants required for good health, including access to quality treatment and care, education, employment, housing, and other opportunities for critical economic growth and investments in wealth-generating initiatives that are associated with privilege and social mobility. These findings are consistent with the conceptualizations of structural racism as influencing health across the life course through multiple and intersecting pathways outlined by previous scholars [9,37,38,39].

Notably, the findings reveal that structural racism has the ability to transcend from structural/macro-level drivers to impact health at the individual level. These findings illuminate how structural-level factors can have micro-level influences at an individual level and can influence one’s chronic disease journey and progression. These findings are consistent with other studies that have emphasized the physical embodiment of one’s social and ecological context has far reaching implications on health and wellbeing [7,40,41,42,43]. Understanding the impacts of structural racism on health and wellbeing requires understanding the broader way in which non-medical factors influence health. This requires moving outside of the biomedical sphere of examining single-issue causes of disease and expanding the scope to look at the environments in which people live that generate poor health and disease.

The findings from this study align with previous research, which has identified unequal access to medical care and treatment as a critical structural racism pathway to health [9,44]. This study found that structural racism impacts and disrupts access to healthcare through three pathways, including being denied treatment or dismissed, refusal to access care due to fear and distrust in the healthcare system, and the lack of or limited access to healthcare and preventative treatment. These findings align with existing literature, which has widely documented First Nations peoples being dismissed, denied treatment, or receiving substantially lower medical care when accessing healthcare in Canada [30,45,46,47,48,49,50,51,52]. The systematic exclusion of First Nations peoples from the healthcare system is one of the ways in which structural racism maintains and reinforces health inequities.

Furthermore, the use of law as an essential tool of structural racism to reinforce and sustain structural racism against First Nations in Canada was evident in the findings. These findings support the arguments of other scholars who describe law as being an essential tool of structural racism [13]. It is clear that the pervasiveness of structural racism, which was described by many participants to exist across all systems and sectors of Canadian society, could not have occurred without the use of colonial laws and policy as a fundamental instrument. The results suggest that the harmful and paternalistic impact of colonial laws and the structures of the Indian Act have played a leading role in upholding colonialism and deeply embedding structural racism into all systems and sectors in society, along with shaping the everyday lives of First Nations peoples.

### 4.1. Limitations

There are limitations of this study that should be considered. Firstly, a majority of the participants interviewed (18 out of 25) were located in Western Canada, particularly the Pacific and Prairie regions, and a limited number of participants were from the Northern or Atlantic regions. Recognizing the heterogeneity within Indigenous communities and diversity of knowledge, perspectives, and experiences across each community and region in Canada, caution should be used when interpreting the results, as they may not be reflective of the perspectives and experiences of all First Nations peoples, particularly those living in the Northern or Atlantic regions of Canada. Secondly, the limited feasibility to conduct in-person interviews due to public health restrictions and social distancing requirements imposed during the COVID-19 pandemic was a limitation. As data collection primarily occurred during the COVID-19 pandemic, in-person interviews were not feasible; thus, all interviews were conducted via telephone or video conference. While there were several benefits to conducting remote interviews, such as increased access to participants located across Canada and convenience for participants to conduct interviews at their preferred location, this may have had unintentional implications when recruiting participants [53]. While in-person interviews were preferred, conducting telephone or video conference interviews may have hindered the development of greater rapport and trust with participants. Furthermore, this may have created barriers for participation for FNLECCs due to preferences to not engage in remote interviewing due to a historical lack of trust in researchers. Therefore, the inability to conduct in-person interviews is a limitation to this study that should be considered.

### 4.2. Recommendations for Future Research

Future research on structural racism and its impact on chronic disease and the health of First Nations is required to further advance our understanding of this complex relationship. Future research should examine how structural racism influences specific chronic conditions to provide a more in-depth understanding of its effect on distinct conditions [1,2,3]. An examination of how structural racism impacts heart disease, cancer, tuberculosis, arthritis, asthma, or other chronic diseases would create opportunities to better understand this complex relationship and may uncover potential ways to target and address the impacts of structural racism on specific conditions. Furthermore, while this study focused specifically on examining the impact of structural racism on the health of First Nations peoples in Canada, less is known about how structural racism impacts Inuit and Métis communities specifically. Given that First Nations, Inuit, and Métis peoples have unique experiences, barriers, and challenges in accessing healthcare, future research exploring each population’s distinct experience with structural racism is needed in order to successfully develop interventions to adequately mitigate and address structural racism for each population. Future research into structural racism on the health of urban First Nations peoples compared to rural/remote First Nations communities may yield more insights into the unique experiences and interactions with structural racism within these environments.

As this study explored structural racism and health from a macro perspective, across multiple domains, it is recommended that future research be undertaken to further uncover the influence of structural racism within each domain and the potential impact on the health of First Nations. New evidence is required to better understand how structural racism within particular domains, for example, domains which are not traditionally viewed as being directly health-related, such as the educational system, justice system, or child welfare system, influence First Nations health and chronic disease. Such research would provide a deeper understanding of the interconnected ways these systems reinforce and sustain structural racism across domains and shed light on interconnected associations between different systems and how they interact and influence health outcomes.

## 5. Conclusions

This study explored the complex and intersecting ways in which structural racism can influence chronic disease outcomes and the overall health and wellbeing of First Nations in Canada. The findings demonstrate that the ways in which structural racism manifests across Canadian society and its pervasiveness across all systems and domains has a role to play in negatively influencing the chronic disease outcomes and the overall health of First Nations peoples. Structural racism was found to influence health through multiple and interacting pathways ranging from systems of failure, harm, and indifference to increasing risk factors for chronic conditions. Recognizing how structural racism shapes our environments may help to catalyze a shift in our collective understanding of the impact of structural racism on health. Structural racism creates an ecosystem that impacts health and chronic disease at the individual level, thus illuminating how the structures of our society have very real health consequences at the individual level. The findings from this study may help to broaden our understanding of the scale of structural racism in shaping the health and wellbeing of First Nations peoples and stimulate attention and action towards dismantling it.

## Figures and Tables

**Table 1:** Themes and Subthemes.

Theme	Subtheme
Multiple and intersecting pathways	Web of inequities and interacting systems of burden Compounding weight
Systems of failure, harm, and indifference	Failure by the system to help Lack of accountability for death and harm
Impacts on access to healthcare	Denied treatment or dismissed Fosters fear and distrust in the healthcare system: will not access Lack of or limited access to healthcare and preventative treatment
Colonial legislation and policies of structural deprivation	Colonial paternalism via the Indian Act: root causes of chronic disease and poor health Resource allocation: chronic underfunding of essential services
Increased risk factors for chronic disease and poor health	Shaping critical social determinants of health Hyper-surveillance: increased risk for interaction with the system Colonial burden and trauma: unhealthy coping mechanisms
Cumulative structural burden leading to individual level health outcomes	Chronic stress and elevated allostatic load Burnout and poor mental health

## Data Availability

The data sets analyzed during the current study are not publicly available due to confidentiality concerns.
